# Morphological, biochemical and molecular characterization of short-day tropical Indian garlic (*Allium sativum* L.)

**DOI:** 10.1016/j.heliyon.2024.e37553

**Published:** 2024-09-06

**Authors:** Karishma Pasupula, Priyanka Verma, Masochon Zimik, Charanjit Kaur, Sujata Vasudev, Anil Khar

**Affiliations:** Division of Vegetable Science, ICAR-Indian Agricultural Research Institute, New Delhi, 110012, India

**Keywords:** Genetic diversity, Yield, Dry matter, Antioxidants, Molecular markers, SSR

## Abstract

Garlic, an asexually propagated bulbous crop, displays a wide diversity based on its morphological traits and biochemical compositions. This study investigated the genetic variability of Indian garlic through morphological, biochemical, and molecular markers. Twenty-nine genotypes along with three Allium species as outgroup were included in the present study. Observations were recorded on 14 quantitative traits, 17 qualitative traits, and 9 biochemical traits in fresh garlic. Significant variability was observed among genotypes for different characters. All the morphological and biochemical traits showed higher phenotypic coefficient of variation (PCV) than genotypic coefficient of variation (GCV) revealing the role of environment in trait expression. High to moderate heritability and genetic advance as percent mean were recorded for different traits except dry matter and Total Soluble Solids (TSS). Correlation analysis revealed the highest positive correlation between total yield, marketable yield, Ferric Reducing Antioxidant Potential (FRAP) and 2,2-diphenyl-1-picrylhyrazyl (DPPH). Cluster analysis differentiated all the genotypes into three major clusters based on morphological and biochemical traits. 214 Simple Sequence Repeats (SSRs) were screened and nine markers exhibited polymorphism. Cluster analysis using molecular markers revealed 4 distinct clusters. The observations from this study will help in the identification of diverse garlic germplasm for its efficient management and duplicate identification of germplasm resources.

## 1 Introduction

Garlic (*Allium sativum* L.), belonging to the family *Amaryllidaceae,* is the second most important bulbous vegetable crop valued throughout the world for its usage as a spice and condiment. Other species belonging to this family include onion (*Allium cepa* L.), garlic chives (*A. tubersoum* L.), shallot (*A. ascalonicum* L.), leek (*A. ameploprasum* L.), welsh onion (*A. fistulosum* L.*)* and chive (*A.schoenoprasum* L.). *A. longicuspis* is considered to be the wild ancestor of garlic [1]. *A. sativum* is a diploid species with 2n = 16 [[2], [3], [4]]. The total world production of garlic is 28 million tonnes with a recorded land area of 1.6 million ha. China is the world's leading garlic producer followed by India [5]. The Indian states of Madhya Pradesh, Rajasthan, Uttar Pradesh and Gujarat are the major garlic-growing states with a countrywide total production of 3.1 million metric tons [6].

Garlic is an asexually propagated, erect annual herb that can reach up to a height of 75–90 cm and is grown usually as a winter crop in the *rabi* season [7] and cultivated mainly for its underground bulbs. Compared to other bulb crops, garlic has a higher nutritive value and is rich in vitamins, proteins, sugars, fat, phosphorus, potassium, iodine, calcium, silicon, and fiber. The pungent flavor of garlic makes it amenable to be used as a spice, flavoring agent, and for the seasoning of foods. Garlic possesses a wide range of sulfur and phenolic compounds having a beneficial effect on human health [[8], [9], [10]]. These sulfur compounds have attributed to garlic its medicinal properties such as antioxidant, antiviral, antifungal, antiprotozoa, antibacterial, and anticancer properties [11]. Although garlic is vegetatively propagated, it has shown wide phenotypic plasticity, diversity in morphological traits and biochemical compositions between and within ecotypes [[12], [13], [14]]. In any crop breeding program, diversity in plant genetic resources in the given breeding population plays a crucial role in determining the rate of genetic gain [15]. It provides an opportunity for developing new and improved cultivars that have both farmer-preferred traits such as yield potential and breeder-preferred traits like biotic and abiotic resilient crops. Morphological characterization along with molecular markers can be used effectively to analyze genetic diversity. Morphological traits are useful in assessing genetic divergence as a preliminary tool for classifying germplasm into groups before their characterization using more precise technology. In garlic, studies of characterization and diversity analysis based on morphological, biochemical, and molecular markers have been carried out [[16], [17], [18], [19], [20], [21]]. However, the estimation of diversity from these phenotypic and genotypic data might correspond differently and hence an accurate analysis cannot be obtained. The aim of the study was threefold 1) to assess the diversity of garlic genotypes based on morphology and biochemical parameters 2) to augment the scarce genomic resources in garlic by cross-amplification of SSR makers from onion, garlic, and related spp. and 3) to examine whether morphological and biochemical-based classification aligns or reveals similar patterns of genomic differentiation as in SSR markers.

## 2 Materials and methods

### 2.1 Plant materials

Twenty-nine Indian garlic genotypes were used in this study. Out of the 29 genotypes, six were landraces, 10 clonal selections, and 13 commercially released varieties. Seven varieties (G1, G41, G50, G189, G282, G323 and G386) have been released by the National Horticultural Research and Development Foundation (NHRDF); GG2 and GG4 by Junagarh Agricultural University (JAU, Gujarat); Bhima Omkar and Bhima Purple from Directorate of Onion and Garlic Research (DOGR, Maharashtra); Godavari and Phule Baswant from Mahatma Phule Krishi Vishvavidyalaya (MPKV, Maharashtra) (Supplementary Table 1). Most of the genotypes are white coloured except PGS208, Phule Bawant, Godavari and Bhima Purple which are purple coloured. Garlic genotypes were grown at the Research Farm of the Division of Vegetable Science, Indian Agricultural Research Institute, New Delhi during 2019–20 situated at 228.61 m above sea level, with 28.08 °N latitude and 77.12 °E longitude. The location has sub-tropical and semi-arid type climate with alluvial soils. The experiment was laid out in a completely randomized block design with three replications. Spacing was 10 cm plant to plant and 15 cm row to row with 20 rows of 2-m length. All the recommended packages of practices were followed for raising the garlic crop. Bulbs were harvested in April and after one month of curing, bulbs were used for biochemical estimation.

#### 2.1.1 Morphological characterization

Fourteen quantitative traits *viz*., plant height, number of leaves, leaf length, leaf width, pseudostem length, pseudostem width, average bulb weight, equatorial diameter, polar diameter, neck thickness, number of cloves, 10 clove weight, total weight, and marketable weight were recorded. Similarly, seventeen qualitative traits *viz*., the density of leaves, foliage attitude, leaf intensity, leaf shape, pseudostem anthocyanin, flowering stem, bulb shape in longitudinal section, the position of cloves at the tip of the bulb, the position of root disc, shape of bulb base, compactness of cloves, ground color of dry external scales, anthocyanin stripes on dry external scales, external cloves, skin adherence of external dry scales, color of clove scale, the color of clove flesh for each of the 29 genotypes were recorded. The observation was recorded as per the DUS guidelines for onion and garlic [22]. Observations were recorded on ten randomly selected samples of each replication excluding the border rows. The average values for each replication were used for statistical analysis.

#### 2.1.2 Biochemical characterization

Garlic bulbs of each genotype harvested after maturity were estimated for the following biochemical parameters. The dry matter percent in clove was estimated by a gravimetric method [23]; Total soluble solids (TSS) by using a hand refractometer; Total phenolic content (TPC) was extracted and measured by spectrophotometry at 750 nm [24], whereas Total flavonoid content (TFC) was determined and absorbance was noted at 510 nm [25]. As for the Total antioxidant capacity, it was determined via Cupric Reducing Antioxidant Capacity (CUPRAC) [26]; Ferric Reducing Antioxidant Potential (FRAP) [27], and DPPH assay [28,29]. Absorbance for CUPRAC, FRAP, and DPPH was recorded against blank spectrophotometrically at different wavelengths i.e., 450 nm, 593 nm, and 517 nm respectively and results were expressed as mol Trolox/g using molar absorptivity of Trolox. Allicin content was assessed [24]and estimated by absorbance at 412 nm and Pyruvic acid content was determined [30] and absorbance noted at 515 nm.

#### 2.1.3 Statistical analysis

To assess the genetic diversity, analysis of variance (ANOVA) for 14 morphological traits and 9 biochemical traits was calculated using PROC ANOVA/GLM. For individual basic statistics, PROC UNIVARIATE was used. The results obtained in the morphological characterization studies were evaluated by performing variance analysis in the SAS version 9.3. Estimation of GCV, PCV, heritability, genetic advance, and genetic advance as percent of mean was done using R software. Diversity analysis was done by subjecting the morphological and biochemical data to Mahalanobis D2 statistics and the grouping pattern of the genotypes was estimated by Principal component analysis (PCA) in software R software on the basis of correlation coefficient between two genotypes.

### 2.2 Molecular characterization

Cloves of all the genotypes were sown and grown for 20–25 days under net-house conditions. DNA was extracted from the leaves of all the genotypes following the CTAB method of isolation [31]. The quality and concentration of DNA were quantified using a Nanodrop spectrophotometer and the integrity was examined by using 0.8 % agarose gel electrophoresis.

#### 2.2.1 Genetic diversity assessment using SSR markers

A total of 214 SSR primers which were sourced from reported onion, garlic, and other Allium spp. markers were used in the study [20], [[32], [33], [34]]. Out of these markers, 116 showed monomorphic bands and 9 were polymorphic (Supplementary Table 2). These 9 polymorphic markers were assayed against thirty-two diverse garlic genotypes, including outgroup viz., *A. tuberosum* L., *A. cepa* L., and *A. fistulosum* L. The PCR reaction was performed in a reaction volume of 10 μl containing 5 μl 2× OnePCR™ (Gene Direx), 1 μl template DNA, 1 μl of specific primer (10 p.m./μl) and volume made up with nuclease-free water. PCR amplification was performed with initial denaturation of 95^o^C for 3 min followed by 35 cycles at 95^o^C for 30 s, 55^o^C for 30s, 72^o^C for 1 min, and the final extension at 72^o^C for 5 min before cooling at 4^o^C. The amplified PCR products were run on 4 % agarose gel stained with ethidium bromide and visualized under a UV transilluminator.

#### 2.2.2 Data analysis

Polymorphic information content (PIC) was calculated to determine the informativeness and discriminatory power of the microsatellite markers using the CERVUS software [35] where P_i_ is the frequency of the *i*th allele in the set of the garlic genotypes under study. Genetic dissimilarity between two genotypes i and j was calculated according to Jaccard's dissimilarity index by using the formula dij = b + c/a + (b + c) where *dij* is the dissimilarity between units *i* and *j*, *xi*, *xj* are variable values for units *i* and *j*, *a* is number of variables where *xi* = presence and *xj* = presence, b is number of variables where *xi* = presence and *xj* = absence and c denotes number of variables where *xi* = absence and *xj* = presence. Tree construction was done by using hierarchical clustering using UPGMA (Unweighted pair group method with arithmetic mean) analysis. All the analysis was performed by using software DARwin [36].

## 3 Results and discussion

### 3.1 Morphological characterization

The mean value for plant height ranged from 61.32 cm (G50) to 84.25 cm (PGS204). Bhima Purple (8.20) had the maximum number of leaves per pseudostem whereas PGS215 (6.33) had the minimum number of leaves (Table 1). The length of the leaves ranged from 38.98 cm (Bhima Purple) to 49.18 cm (PGS-204). In terms of leaf width, the highest value was measured in Phule Baswant (2.22 cm), while the lowest value (1.61 cm) was observed in PGS-215. Among 29 genotypes of garlic, the mean value for pseudostem length ranged from 21.15 cm to 37.00 cm, with maximum observed in PGS204 and minimum in G50. The pseudostem width was found to be maximum for G282 (1.20 cm) and the minimum was recorded for PGS200 (0.87 cm). The results were in accordance with the results of other authors [[37], [38], [39], [40]]. The average bulb weight of the 29 genotypes was determined in the range of 21.00–34.80 g, with genotypes G282 and G41 with the highest weight and GG4 with the lowest weight. Significant variations in bulb weight per plant were also reported by Panthee et al. [16] and Sandhu et al. [37]. In terms of the bulb equatorial diameter, genotype PGS202 (4.72 cm) was superior whereas genotype GG4 had the lowest bulb equatorial diameter. For bulb polar diameter, Bhima Omkar (3.57 cm) was found to be superior. Neck thickness ranged from 0.65 cm (PGS211, PGS212) to 0.56 cm (PGS200, PGS215). The average number of cloves per bulb was recorded to be in the range of a maximum of 30.96 in genotype G1 and a minimum of 13.53 in G386. In the case of the 10 clove weight, G386 had the highest weight measuring 25.66 g. The observations were consistent with the results of Sandhu et al. [37] and various other authors [41,42]. The superior genotype for total yield was G282 with a marketable weight of 128.70 q/ha while the superior genotype for marketable yield was also G282 (104.53 q/ha). Similar observations were reported by other researchers [40,42,43].

**Table 1:** Mean performance for different morphological traits in garlic (*Allium sativum* L.).

Genotype	Plant height (cm)	Number of leaves	Leaf length (cm)	Leaf width (mm)	Pseudostem length (cm)	Pseudostem width (cm)	Average bulb weight (g)
PGS200	77.30 ± 1.39^b^	6.86 ± 0.14^jk^	46.47 ± 0.77^bcd^	1.65 ± 0.05^kl^	31.66 ± 1.11^bcd^	0.87 ± 0.04^k^	28.40 ± 1.73^efgh^
PGS201	70.90 ± 1.65^efgh^	7.20 ± 0.11^ghij^	43.25 ± 0.94^ghij^	1.74 ± 0.07^hijkl^	26.86 ± 1.06^hijkil^	0.91 ± 0.03^jk^	31.60 ± 1.92^bcde^
PGS202	78.28 ± 1.29^b^	7.53 ± 0.18^cdefg^	47.66 ± 0.82^abc^	1.98 ± 0.06^bcde^	32.96 ± 1.08^b^	1.11 ± 0.04^abc^	34.80 ± 0.97^ab^
PGS203	77.05 ± 1.87^bc^	6.90 ± 0.12^jk^	47.06 ± 0.98^abc^	1.93 ± 0.07^bcdef^	30.25 ± 1.19^cdef^	0.98 ± 0.04^efghij^	32.13 ± 1.81^abcde^
PGS204	84.25 ± 1.55^a^	6.90 ± 0.13^jk^	49.18 ± 0.80^a^	1.77 ± 0.03^ghijk^	37.00 ± 1.02^a^	0.98 ± 0.02^efghij^	34.06 ± 2.39^abc^
PGS205	72.88 ± 2.25^cdef^	7.13 ± 0.12^ghij^	43.68 ± 0.89^efghi^	1.82 ± 0.04^fghij^	29.64 ± 1.01^cdefg^	0.94 ± 0.02^hijk^	30.33 ± 1.11^cdefg^
PGS206	77.83 ± 1.22^b^	7.16 ± 0.16^ghij^	47.84 ± 0.75^ab^	1.99 ± 0.05^bcde^	31.33 ± 0.72^bcde^	1.13 ± 0.04^ab^	32.86 ± 1.48^abcd^
PGS207	75.36 ± 1.85^bcd^	6.86 ± 0.14^jk^	46.40 ± 0.69^bcd^	1.82 ± 0.05^fghij^	32.07 ± 0.83^bc^	0.91 ± 0.04^jk^	31.26 ± 1.45^bcde^
PGS208	70.33 ± 1.19^efghi^	7.63 ± 0.12^bcdef^	40.90 ± 0.96^jk^	1.95 ± 0.04^bcdef^	29.12 ± 0.94^efghi^	0.96 ± 0.02^ghijk^	26.33 ± 0.93^gh^
PGS209	72.95 ± 1.54^cdef^	7.66 ± 0.16^bcde^	42.70 ± 0.93^hij^	2.05 ± 0.05^bc^	31.60 ± 0.98^bcde^	1.06 ± 0.03^bcdef^	32.33 ± 1.66^abcde^
PGS210	65.68 ± 1.54^jk^	6.93 ± 0.15^ijk^	44.14 ± 1.05^defgh^	1.85 ± 0.05^efghi^	21.54 ± 0.78^n^	0.92 ± 0.03^ijk^	33.73 ± 1.57^abc^
PGS211	68.90 ± 1.62^fghijk^	7.23 ± 0.14^fghij^	44.03 ± 1.12^defghi^	1.97 ± 0.04^bcde^	24.50 ± 0.80^lm^	0.93 ± 0.03^hijk^	31.13 ± 1.17^bcde^
PGS212	67.99 ± 1.76^ghijk^	7.33 ± 0.22^efghi^	42.32 ± 1.20^hij^	1.72 ± 0.05^ijkl^	24.61 ± 1.16^lm^	0.91 ± 0.02^jk^	26.40 ± 0.90^gh^
G1	66.61 ± 1.14^hijk^	6.63 ± 0.14^kl^	43.49 ± 0.91^fghi^	1.81 ± 0.05^fghij^	23.41 ± 0.70^mn^	0.98 ± 0.03^efghij^	26.86 ± 1.34^fgh^
G41	77.90 ± 1.52^b^	7.03 ± 0.16^hijk^	45.95 ± 0.95^bcdef^	1.89 ± 0.05^defgh^	31.75 ± 1.05^bc^	0.99 ± 0.02^efghij^	34.86 ± 1.92^ab^
G50	61.32 ± 1.32^l^	7.03 ± 0.13^hijk^	40.92 ± 0.85^jk^	1.78 ± 0.04^ghijk^	21.15 ± 0.86^n^	0.96 ± 0.02^fghijk^	25.00 ± 1.13^hi^
G189	71.68 ± 1.13^defg^	7.03 ± 0.16^hijk^	46.30 ± 0.85^bcd^	1.98 ± 0.05^bcde^	24.80 ± 0.66^klm^	1.12 ± 0.04^abc^	36.13 ± 1.86^a^
G282	74.00 ± 1.30^bcde^	7.76 ± 0.16^bcd^	46.45 ± 0.74^bcd^	1.99 ± 0.03^bcde^	28.75 ± 1.11^fghi^	1.20 ± 0.04^a^	34.86 ± 2.43^ab^
G323	70.61 ± 2.05^efgh^	7.20 ± 0.14^ghij^	46.16 ± 1.14^bcde^	1.99 ± 0.07^bcde^	26.09 ± 0.90^jkl^	1.01 ± 0.03^defghi^	30.20 ± 1.47^cdefg^
G386	72.19 ± 1.12^defg^	7.73 ± 0.12^bcde^	45.24 ± 0.83^cdefg^	1.88 ± 0.03^efgh^	30.36 ± 0.90^cdef^	0.90 ± 0.03	31.86 ± 1.59^bcde^
GG2	74.18 ± 1.20^bcde^	7.70 ± 0.17^bcde^	45.75 ± 0.83^bcdefg^	1.90 ± 0.06^cdefg^	29.15 ± 0.71^defgh^	0.98 ± 0.03^efghij^	26.00 ± 0.93^h^
GG4	67.16 ± 1.93^hijk^	7.03 ± 0.17^hijk^	42.60 ± 0.93^hij^	1.69 ± 0.05^jkl^	27.90 ± 0.71^fghij^	0.90 ± 0.03^jk^	21.00 ± 1.39^i^
Bhima Omkar	66.10 ± 1.27^ijk^	7.96 ± 0.14^ab^	41.53 ± 14.8^ij^	1.99 ± 0.05^bcde^	27.55 ± 0.60^ghij^	1.04 ± 0.03^bcdefg^	33.20 ± 1.43^abcd^
Bhima Purple	64.56 ± 1.38^kl^	8.20 ± 0.12^a^	38.98 ± 0.96^k^	2.07 ± 0.04^ab^	28.01 ± 0.87^fghij^	1.10 ± 0.03^abcd^	29.13 ± 1.20^defgh^
Godavari	69.23 ± 1.57^fghij^	7.36 ± 0.21^defgh^	42.66 ± 1.66^hij^	2.04 ± 0.06^bcd^	25.87 ± 0.94^jklm^	1.07 ± 0.04^bcde^	29.13 ± 0.78^defgh^
Phule Baswant	70.15 ± 1.33^efghi^	7.83 ± 0.17^abc^	42.55 ± 0.97^hij^	2.22 ± 0.07^a^	27.31 ± 0.80^ghijk^	1.02 ± 0.04^cdefgh^	30.80 ± 1.27^bcdef^
PGS215	74.32 ± 2.70^bcde^	6.33 ± 0.15^l^	46.25 ± 0.84^bcd^	1.61 ± 0.04^l^	31.91 ± 0.81^bc^	0.90 ± 0.02^jk^	25.66 ± 1.00^h^
PGS216	72.14 ± 1.41^defg^	7.70 ± 0.13^bcde^	45.54 ± 0.66^bcdefg^	1.85 ± 0.03^efghi^	26.61 ± 1.01^ijkl^	1.02 ± 0.03^cdefgh^	31.46 ± 1.28^bcde^
PGS217	67.28 ± 1.36^hijk^	7.13 ± 0.11^ghij^	44.29 ± 0.62^defgh^	1.90 ± 0.04^cdefg^	21.79 ± 0.95^n^	0.99 ± 0.02^efghij^	26.00 ± 1.15^h^
CV (%)	11.95	11.19	35.06	15.91	17.72	19.21	19.03
R square	0.27	0.26	0.05	0.17	0.38	0.19	0.30

Genotype	Equatorial diameter (cm)	Polar diameter (cm)	Neck thickness (cm)	Number of cloves	10 Clove weight (g)	Total weight (q/ha)	Marketable weight (q/ha)
PGS200	4.29 ± 0.08^defgh^	3.28 ± 0.07^defghi^	0.56 ± 0.02^e^	25.86 ± 1.20^cde^	15.33 ± 0.64^fghij^	80.13 ± 6.23^ijk^	61.96 ± 8.77^ghijk^
PGS201	4.48 ± 0.09^abcd^	3.32 ± 0.07^cdefg^	0.61 ± 0.02 ^abcde^	24.60 ± 1.26^cdefg^	18.80 ± 1.33^bcde^	80.63 ± 13.73^hijk^	59.56 ± 13.34^ghijk^
PGS202	4.72 ± 0.04^a^	3.52 ± 0.07^ab^	0.63 ± 0.02^abcde^	22.80 ± 1.11^defgh^	23.33 ± 0.67^a^	106.40 ± 3.51^cde^	86.20 ± 1.47^abcde^
PGS203	4.41 ± 0.11^cdef^	3.30 ± 0.09^cdefg^	0.64 ± 0.02^abc^	21.93 ± 1.35^fgh^	20.33 ± 1.01^bc^	91.60 ± 7.39^efghi^	74.40 ± 8.82^defgh^
PGS204	4.54 ± 0.12^abc^	3.38 ± 0.06^abcdef^	0.61 ± 0.02 ^abcde^	25.53 ± 1.54^cdef^	17.80 ± 0.82^cdef^	115.13 ± 22.26^abcd^	88.23 ± 16.88^abcde^
PGS205	4.42 ± 0.05^cde^	3.24 ± 0.04^fghij^	0.58 ± 0.02 ^abcde^	28.13 ± 1.93^abc^	15.73 ± 1.01^fghi^	80.80 ± 17.76^hijk^	55.20 ± 11.68^ijkl^
PGS206	4.62 ± 0.09^abc^	3.34 ± 0.05^bcdef^	0.62 ± 0.02 ^abcde^	29.80 ± 1.71^ab^	19.06 ± 1.39^bcde^	84.86 ± 5.57^fghi^	66.00 ± 6.63^fghij^
PGS207	4.49 ± 0.09^abcd^	3.23 ± 0.04^fghij^	0.61 ± 0.02 ^abcde^	22.73 ± 0.58^defgh^	20.00 ± 0.97^bcd^	125.60 ± 6.90^ab^	99.66 ± 6.12^ab^
PGS208	3.81 ± 0.08^kl^	3.26 ± 0.06^defghij^	0.57 ± 0.02^cde^	20.26 ± 1.05^h^	15.06 ± 0.38^ghij^	83.50 ± 9.47^ghij^	69.70 ± 12.97^efghi^
PGS209	4.46 ± 0.06^bcd^	3.36 ± 0.07^bcdef^	0.63 ± 0.03^abcde^	25.06 ± 2.02^cdefg^	17.46 ± 0.91^defg^	111.46 ± 7.77^abcd^	84.50 ± 1.23^bcdef^
PGS210	4.38 ± 0.09^cdefg^	3.34 ± 0.05^bcdef^	0.60 ± 0.03 ^abcde^	22.86 ± 0.91^defgh^	20.13 ± 0.95^bc^	118.86 ± 4.31^abc^	88.53 ± 2.93^abcd^
PGS211	4.17 ± 0.06^efghi^	3.22 ± 0.08^fghij^	0.65 ± 0.03^ab^	26.33 ± 0.88^bcd^	15.60 ± 0.86^fghi^	101.36 ± 14.01^cdef^	83.83 ± 9.95^bcdef^
PGS212	3.94 ± 0.06^ijk^	3.44 ± 0.05^abcde^	0.65 ± 0.02^a^	15.66 ± 0.76^i^	19.60 ± 0.66^bcde^	98.66 ± 6.54^defg^	80.80 ± 5.25^cdef^
G1	4.16 ± 0.10^fghi^	3.08 ± 0.06^j^	0.60 ± 0.05 ^abcde^	30.96 ± 1.58^a^	11.53 ± 0.68^l^	64.00 ± 5.90^kl^	49.13 ± 6.24^jkl^
G41	4.54 ± 0.08^abcd^	3.49 ± 0.04^abc^	0.62 ± 0.01 ^abcde^	30.00 ± 1.24^a^	15.73 ± 1.12^fghi^	109.76 ± 11.66^bcd^	96.80 ± 10.19^abc^
G50	4.02 ± 0.08^ijk^	3.10 ± 0.06^hij^	0.60 ± 0.02 ^abcde^	29.80 ± 1.61^ab^	11.26 ± 0.87^l^	66.00 ± 10.95^jkl^	46.80 ± 7.61^kl^
G189	4.69 ± 0.10^ab^	3.31 ± 0.06^cdefg^	0.63 ± 0.02^abcd^	29.93 ± 1.89^ab^	15.06 ± 0.61^ghij^	112.10 ± 0.00^abcd^	94.93 ± 5.56^abc^
G282	4.57 ± 0.13^abc^	3.31 ± 0.08^cdefg^	0.59 ± 0.02 ^abcde^	22.86 ± 1.34^defgh^	20.46 ± 1.31^b^	128.70 ± 14.97^a^	104.53 ± 12.21^a^
G323	4.17 ± 0.11^fghi^	3.31 ± 0.07^cdefg^	0.63 ± 0.03^abcd^	27.93 ± 1.90^abc^	14.20 ± 0.83^ijk^	108.40 ± 9.03^bcde^	87.86 ± 8.16^abcde^
G386	4.06 ± 0.11^ijh^	3.45 ± 0.08^abcd^	0.63 ± 0.01 ^abcde^	13.53 ± 0.86^i^	25.66 ± 1.17^a^	114.46 ± 19.80^abcd^	99.66 ± 19.43^ab^
GG2	3.87 ± 0.08^jk^	3.22 ± 0.09^fghij^	0.61 ± 0.01 ^abcde^	24.66 ± 1.29^cdefg^	12.93 ± 0.66^jkl^	78.43 ± 7.62^ijk^	60.93 ± 2.76^ghijk^
GG4	3.59 ± 0.09^l^	3.13 ± 0.07^ghij^	0.60 ± 0.02 ^abcde^	21.60 ± 0.94^gh^	11.73 ± 0.82^kl^	60.30 ± 2.76^l^	39.06 ± 6.17^l^
Bhima Omkar	4.46 ± 0.08^bcd^	3.57 ± 0.08^a^	0.61 ± 0.01 ^abcde^	22.33 ± 0.87^efgh^	19.46 ± 1.09 ^bcde^	113.13 ± 5.92^abcd^	96.63 ± 2.43^abc^
Bhima Purple	4.07 ± 0.08^hij^	3.29 ± 0.09^defgh^	0.58 ± 0.02 ^abcde^	20.00 ± 0.93^h^	17.06 ± 0.54^efgh^	104.03 ± 13.87^cde^	86.03 ± 6.46^abcde^
Godavari	4.13 ± 0.07^hi^	3.45 ± 0.06^abcd^	0.61 ± 0.03 ^abcde^	24.00 ± 1.11^defg^	15.93 ± 1.06^fghi^	97.96 ± 16.17^defgh^	75.76 ± 6.72^defg^
Phule Baswant	4.11 ± 0.08^hij^	3.36 ± 0.04^bcdef^	0.57 ± 0.02^bcde^	23.06 ± 1.10^defgh^	16.20 ± 0.94^fghi^	98.00 ± 10.90^defgh^	82.16 ± 7.62^bcdef^
PGS215	4.05 ± 0.07^hijk^	3.09 ± 0.07^ji^	0.56 ± 0.02^de^	24.20 ± 1.14^defg^	14.86 ± 0.63^ijh^	76.76 ± 19.66^ijkl^	55.90 ± 19.54^hijkl^
PGS216	4.45 ± 0.06^bcd^	3.26 ± 0.06^defghij^	0.61 ± 0.02 ^abcde^	22.13 ± 0.55^fgh^	20.00 ± 0.78^bcd^	111.43 ± 5.51^abcd^	84.16 ± 7.81^bcdef^
PGS217	4.15 ± 0.07^ghi^	3.26 ± 0.06^efghij^	0.56 ± 0.01^de^	22.13 ± 1.46^fgh^	14.40 ± 0.72^ij^	85.63 ± 7.31^fghi^	61.26 ± 12.09^ghijk^
CV (%)	8.03	8.02	16.83	20.90	20.75	11.06	14.80
R square	0.42	0.21	0.07	0.41	0.50	0.88	0.83

### 3.2 Biochemical characterization

The dry matter content in the cultivated garlic was in the range of 36.00–42.25 % (CV = 2.72 %) (Table 2). A high content of dry matter (>41 %) was noted in the genotypes PGS203, G282, and PGS216. A dry matter concentration of 35.2 percent in garlic cloves was observed by Sandhu et al. [37], which was somewhat lower than our findings. In our study, the TSS content of the garlic genotypes ranged from 36.25 to 42.42 °B. G323 had the highest TSS value closely followed by G386 at 42.38 and GG-4 had the lowest followed by GG2 at 36.99. Our results were consistent with the results of Bhusal et al. [44] who also reported the TSS content within the range of 38.10–47.20°B. Variability in garlic genotypes for TSS and dry matter content was also reported by other researchers [43,45,46]. In terms of total phenolic content, the total phenolic content (TPC) varied greatly among all garlic bulb samples, from 25.75 to 75.00 mg/100 g with an average of 44.00 mg/100 g FW. The highest Total Phenolic content (TPC) was found in PGS202 (75.00 mg GAE/100 g) and the lowest was found in G41 (28.33 mg GAE/100 g). TPC of 37.60 ± 2.3 mg GAE/100 g which was similar to the results reported by Othman et al. [47]. On the other hand, a higher phenol content of cloves with an average of 83.58 mg/100 g FW was also observed [48]. Variability for total phenol content in garlic genotypes was reported by other studies [44,[49], [50], [51]]. The level of the total flavonoid content varied from 19.00 to 129.16 mg QE/100 g. The highest value was recorded in the genotype PGS202 and the lowest value was recorded in PGS212 (19.00 mg). Variations in total flavonoid content among garlic genotypes have been earlier reported in the literature [52,53].

**Table 2:** Mean performance of different biochemical traits in garlic (*Allium sativum* L.).

Genotype	Dry matter (%)	TSS (°B)	Total phenolics content (mg GAE/100 g FW)	Total flavonoid content (mgQE/100 g FW)	CUPRAC (μmolTrolox/g FW)	FRAP (μmolTrolox/g FW)	DPPH (μmolTrolox/g FW)	Allicin (mg/100 g)	Pyruvic acid (μMol/ml)
PGS200	39.73^cdefgh^	39.94^defghi^	66.58^c^	67.50^g^	3.02^ef^	1.98^b^	1.75^e^	3.27^p^	26.84^f^
PGS201	37.33^ijklm^	39.85^efghi^	66.33^c^	85.67^d^	2.72^g^	1.81^e^	1.54^k^	3.25^p^	30.65^c^
PGS02	41.59^abc^	38.74^ghijk^	75.00^a^	129.17^a^	2.28^j^	0.91^o^	1.35^p^	5.48^gh^	26.91^f^
PGS203	41.91^ab^	38.41^ijkl^	47.00^g^	82.17^e^	3.06^e^	1.82^de^	1.59^j^	5.37^hi^	30.45^c^
PGS204	39.75^cdefgh^	40.84^abcde^	53.00^e^	61.83^hi^	1.49^p^	1.85^d^	1.70^fg^	6.16^e^	20.14^mn^
PGS205	36.90^jklm^	38.54^hijkl^	33.42^n^	98.67^b^	2.04^l^	1.76^f^	1.95^b^	5.95^f^	21.40^jk^
PGS206	36.40^lm^	40.51^bcdef^	44.42^h^	33.67^no^	1.38^q^	1.54^i^	1.05^t^	5.07^k^	15.73^p^
PGS207	38.85^fghij^	40.32^bcdefg^	51.33^f^	42.00^k^	1.73^n^	1.74^f^	1.83^d^	3.89^n^	20.95^l^
PGS208	36.84^klm^	38.89^fghijk^	37.41^l^	37.17^m^	1.61^o^	1.36^k^	1.24^s^	5.66^g^	19.73^n^
PGS209	40.35^abcdefg^	41.40^abcde^	32.67^n^	60.33^i^	0.47^r^	1.64^g^	1.40^o^	3.84^n^	9.68^s^
PGS210	40.13^bcdefgh^	41.57^abcd^	31.67^o^	53.17^j^	1.90^m^	1.43^j^	1.71^f^	5.66^g^	25.94^g^
PGS211	39.65^cdefgh^	41.31^abcde^	35.50^m^	31.50^p^	1.88^m^	1.11^n^	1.28^q^	4.76^l^	16.61^o^
PGS212	36.51^lm^	38.08^jkl^	39.67^k^	19.00^t^	1.65^o^	1.60^h^	1.61^i^	5.14^jk^	60.92^a^
G1	41.0^abcde^	40.32^bcdefg^	44.42^h^	34.83^n^	2.03^l^	1.58^hi^	1.55^k^	3.55^o^	24.39^h^
G41	38.93^fghi^	40.49^bcdef^	28.33^p^	27.83^q^	1.89^m^	1.35^k^	1.44^n^	6.68^c^	40.83^b^
G50	39.0^efghi^	38.07^jkl^	46.33^g^	24.83^r^	3.45^c^	1.18^m^	1.55^k^	7.30^a^	24.70^h^
G189	39.39^defgh^	38.49^ijkl^	46.17^g^	24.67^r^	2.47^i^	0.85^p^	1.4^o^	6.72^c^	14.37^q^
G282	42.25^a^	41.78^abc^	68.42^b^	78.17^f^	2.97^f^	2.79^a^	2.18^a^	5.06^k^	21.67^j^
G323	39.61^cdefgh^	42.42^a^	43.08^i^	20.50th	2.24^j^	0.82^p^	1.51^l^	3.26^p^	22.59^i^
G386	38.69^fghijk^	42.38^a^	61.25^d^	32.67^op^	2.97^f^	1.43^j^	1.94^b^	2.70^q^	22.50^i^
GG2	37.10^ijklm^	36.99^lm^	33.67^n^	27.50^q^	1.77^n^	1.24^l^	1.86^c^	6.92^b^	29.79^d^
GG4	36.78^klm^	36.25^m^	41.50^j^	68.00^g^	1.46^p^	0.95^o^	1.64^h^	5.89^f^	26.64^f^
Bhima Omkar	41.07^abcde^	38.60^hijkl^	33.50^n^	53.00^j^	1.44^p^	0.43^s^	1.40^o^	5.18^jk^	20.95^l^
Bhima Purple	41.3^abcd^	40.62^bcde^	30.75^o^	89.33^c^	1.67^o^	0.56^q^	1.46^m^	3.60^o^	21.15^kl^
Godavari	38.37^ghijkl^	41.22^abcde^	33.42^n^	62.67^h^	3.66^b^	0.57^q^	1.50^l^	4.40^m^	13.22^r^
Phule Baswant	40.74^abcdef^	41.88^ab^	25.75^q^	22.33^s^	4.37^a^	0.50^r^	1.26^r^	5.17^jk^	22.41^i^
PGS215	38.14^hijkl^	38.98^fghij^	39.75^k^	60.83^hi^	3.26^d^	1.08^n^	1.69^g^	5.51^gh^	28.82^e^
PGS216	42.06^ab^	40.18^cdefgh^	46.25^g^	81.33^e^	2.60^h^	1.93^c^	2.17^a^	5.28^ij^	20.51^m^
PGS217	36.0^m^	37.27^klm^	38.75^k^	39.17^l^	2.16^k^	1.10^n^	1.83^d^	6.51^d^	29.92^d^
CV (%)	2.72	4.43	1.38	2.15	1.53	1.68	0.51	2.08	1.02
R square	0.82	0.52	0.99	0.99	0.99	0.99	0.99	0.99	1.00

AOA measurement is a reliable indicator for estimating the health-promoting and functional dietary value of vegetables and fruits [54]. In the present study, we used three *in-vitro* assays, namely, Cupric ion reducing antioxidant capacity (CUPRAC), Ferric reducing antioxidant power (FRAP), and 1,1-diphenyl-2 picrylhydrazyl (DPPH) assay. In our study, CUPRAC in fresh garlic ranged from 0.47 to 4.37 μmolTrolox/g, maximum recorded in the genotype Phule Baswant and the lowest value recorded in PGS-209. Ferric Reducing Antioxidant Potential (FRAP) analysis showed that genotype G-282 had the highest value with 2.79 μmolTrolox/g whereas Bhima Omkar had the lowest value of 0.43 μmolTrolox/g. The maximum total DPPH antioxidant activity was recorded in G282 and PGS215 followed by PGS205. Significant differences for antioxidant content by different methods in garlic genotypes were earlier reported by other authors [45,47], and [51]. The concentration of allicin in these genotypes varied from 2.70 (G386) to 7.30 mg/100 g (G50) with a mean value of 5.08. Similar observations on the content of allicin were found in other studies. Allicin content ranging between 1.48 and 5.32 mg/g with a mean of 3.16 mg/g FW was observed [53] in Indian garlic genotypes. Significant differences in allicin content for garlic genotypes were also reported in the literature [8,37], and [55]. The pyruvic acid content was found maximum in PGS212 followed by G41 and PGS201 and ranged from 9.67 to 60.91 μmol/ml. A higher pyruvate content ranging from 60.00 μmol/g to 84.00 μmol/g was observed by Abedi et al. [56]. Whereas Bhusal et al. [44] reported values ranging from 49.67 to 76.35 μmol/ml which were similar to our results. The variability in garlic genotypes for pyruvic acid content was also reported by Akinwande and Olatunde [57].

Evaluation of PCV, GCV, and heredity helps to estimate the contribution of the environment and the genes towards the expression of each trait. In this present study, the PCV was greater than the GCV for traits like plant height, number of leaves, leaf length, leaf width, pseudostem length, pseudostem width, average bulb weight, equatorial diameter, polar diameter, neck thickness, number of cloves, average clove weight, total and marketable weight, suggesting that the environment plays an important role in determining variation in these traits (Table 3). Among all the traits examined, high PCV was observed for marketable weight (29.7), total weight (24.86), and average clove weight (22.79), number of cloves (20.22). Moderate estimates of PCV were observed for pseudostem length (16.28), average bulb weight (15.43), pseudostem width (12.43), and GCV were observed for marketable weight (19.25), average clove weight (18.76), total weight (15.75), number of cloves (15.09), pseudostem length (11.74), average bulb weight (10.05). The high to moderate values of PCV and GCV recorded for the above traits indicate the existence of genetic variability and these traits have reasonable scope for additional characteristic development through selection. Low estimates of GCV and PCV were recorded for plant height (6.06 and 8.49), number of leaves (4.66 and 7.78), leaf length (4.71 and 6.35), leaf width (5.58 and 9.69), equatorial diameter (5.89 and 7.78) and low GCV for pseudostem width (4.9). A high PCV for average clove weight, number of cloves, and leaf length were reported by Sharma and Chauhan [58] whereas higher PCV and GCV estimates were also reported by other authors [37,59].

**Table 3:** Estimation of coefficient of variation and other genetic parameter for different morphological and biochemical traits in garlic (*Allium sativum* L.).

	Source of variation and mean squares			Estimation of genetic parameters				Performance						
Source of Variation	Replication	Genotype	Error	GCV	PCV	$h_{\text{BS}}^{2}$ (%)	GA 5 %	Mean	Min	Max	CV%	SEm (±)	CD 5 %	CD 1 %
df	2	28	56											
PH	65.871*	74.782**	18.194	6.06	8.49	50.90	8.90	71.69	61.32	84.25	5.95	2.46	6.98	9.29
PsL	34.471*	42.779**	10.080	11.74	16.29	51.95	17.42	28.12	21.15	37.00	11.29	1.83	5.19	6.91
PsW	0.075505*	0.020225	0.012965	4.91	12.43	15.58	3.98	1.00	0.88	1.20	11.40	0.07	0.19	0.25
NOL	2.18356**	0.55149**	0.20571	4.67	7.78	35.92	5.75	7.28	6.33	8.20	6.23	0.26	0.74	0.99
LL	93.915	16.827	3.593	4.72	6.35	55.11	7.21	44.54	38.98	49.18	4.26	1.09	3.10	4.13
LW	0.014797	0.055983**	0.022442	5.59	9.68	33.33	6.64	1.89	1.62	2.22	7.91	0.09	0.25	0.33
P	0.117774*	0.044858*	0.024835	2.47	5.36	21.27	2.34	3.31	3.09	3.57	4.76	0.09	0.26	0.34
E	0.148698	0.237031**	0.047236	5.89	7.78	57.29	9.17	4.27	3.59	4.72	5.09	0.13	0.36	0.47
ABW	5.780	40.262**	12.540	10.05	15.43	42.43	13.48	30.25	21.00	36.13	11.71	2.04	5.79	7.71
NOC	14.799	50.509**	10.577	15.09	20.22	55.72	23.20	24.17	13.53	31.27	13.45	1.88	5.32	7.08
10CW	2.671	35.587**	4.871	18.76	22.79	67.76	31.81	17.06	11.27	25.67	12.94	1.27	3.61	4.81
TSS	16.0913*	8.3082**	3.5654	3.16	5.70	30.72	3.60	39.81	36.26	42.42	4.74	1.09	3.09	4.11
TPC	1.64*	502.03**	0.37	29.40	29.44	99.78	60.50	43.98	25.75	75.00	1.39	0.35	1.00	1.33
TFC	4.68*	2298.47**	1.31	51.79	51.83	99.83	106.59	53.43	19.00	129.17	2.15	0.66	1.88	2.50
CUPRAC	0.00068	2.09005**	0.00122	36.87	36.90	99.83	75.89	2.26	0.47	4.37	1.54	0.02	0.06	0.08
FRAP	0.00013	0.85294**	0.00050	39.65	39.69	99.82	81.61	1.34	0.43	2.79	1.66	0.01	0.04	0.05
DPPH	0.000074	0.217023**	0.000057	16.80	16.81	99.86	34.58	1.60	2.71	7.30	0.47	0.00	0.01	0.02
Allicin	0.0043	4.6082	0.0112	24.38	24.47	99.27	50.03	5.08	2.71	7.30	2.08	0.06	0.17	0.23
PA	0.114	264.916**	0.062	38.35	38.37	99.93	78.97	24.50	9.68	60.92	1.02	0.14	0.41	0.54
DM	0.1946	10.6449**	1.1339	4.54	5.29	73.66	8.03	39.19	36.01	42.25	2.72	0.61	1.74	2.32
TW	1458.40*	1044.78**	347.01	15.75	24.86	40.13	20.55	96.83	60.27	128.70	19.24	10.76	30.47	40.56
MW	24.94	951.81**	299.82	19.25	29.70	42.02	25.71	76.57	39.06	104.55	22.62	10.00	28.32	37.70

PH-Plant height; PsL-Pseudostem length; PsW-Pseudostem width; NOL-Number of leaves; LL-Leaf length; LW-Leaf width; P-Polar diameter; E-Equatorial diameter; ABW-Average bulb weight; NON-Number of cloves; 10CW-10 clove weight; TSS-Total soluble solids; TPC-Total phenolic content; TFC-Total flavonoid content; PA-Pyruvic acid; DM-Dry matter, TW-Total weight; MW-Marketable weight.

In terms of biochemical traits, among 29 genotypes of garlic, high GCV and PCV estimates were recorded. Higher values for total flavonoid content (51.79 and 51.83), FRAP antioxidant activity (39.65 and 39.69), pyruvic acid content (38.35 and 38.37), CUPRAC antioxidant activity (36.87 and 36.90), total phenolic content (29.40 and 29.44), allicin content (24.38 and 24.47) were recorded. Moderate coefficient of variation for genotypic and phenotypic levels was recorded for DPPH antioxidant activity (16.80 and 16.81). A low estimate for genotypic and phenotypic coefficient of variation was recorded for dry matter content of fresh garlic (4.54 and 5.29) and TSS (3.16 and 5.70). PCV and GCV estimates in garlic for biochemical trait has been reported by Sadhu et al. [37].

Heritability indicates the possibility and extent to which improvement is possible through selection. The genotypic coefficient of variance together with heritability estimates would provide the most accurate depiction of the amount of progress that may be predicted from selection [60]. In the present investigation, broad sense heritability (*H*^*2*^*)* was estimated and it ranged from 15.58% to 67.76 % for morphological traits indicating low to moderate heritability (Table 3). Moderate heritability estimates were recorded for the trait 10 clove weight (67.76 %), equatorial diameter (57.29 %), number of cloves (55.72 %), leaf length (55.11 %), pseudostem length (51.95 %), plant height (50.90 %). The lowest heritability was observed for the marketable weight (42.02 %), average bulb weight (42.43 %), total weight (40.13 %), number of leaves (35.92 %), leaf width (33.33 %), polar diameter (21.27 %) and pseudostem width (15.58 %). Heritability estimates of 0.38–0.95 [61], 0.43–0.95 [62] and 0.32–0.97 [63] were observed in earlier studies which is in contrast to our findings. Fehr [64] suggested that heritability of a trait is determined by the population studied, the environment and the method used. In the present research, high genetic advance as a percent of mean was found high for 10 clove weight (31.81), marketable weight (25.71), average number of cloves (23.20), and total weight (20.55). Moderate genetic advance for pseudostem length (17.43), average bulb weight (13.48). Low genetic advance as a percent of mean was found in equatorial diameter (9.18), plant height (8.9), leaf length (7.21), leaf width (6.64), number of leaves (5.76), pseudostem width (3.99) and polar diameter (2.35). Broad sense heritability was also observed for all biochemical traits i.e., total phenol content, total flavonoid content, antioxidant activity of CUPRAC, FRAP, DPPH, allicin content, and pyruvic acid content. Moderate heritability for dry matter content (73.66 %) and low heritability was recorded for TSS (30.72 %). In the present research, high genetic advance as a percent of the mean was found high for total flavonoid content (106.59), FRAP (81.61), pyruvic acid content (78.97), CUPRAC (75.89), total phenolic content (60.50), allicin content of fresh garlic (50.03) and DPPH (34.58). Low genetic advance as a percent of the mean was found in dry matter content (8.03) and TSS (3.60). High heritability alongwith high genetic advance is a positive trait for breeding high yielding varieties. In our present research, moderate heritability was observed for morphological traits but high heritability for biochemical traits was observed. Hence, selection for biochemical traits can be carried out in the promising genotypes with high values of biochemical traits.

**Fig. 1:**
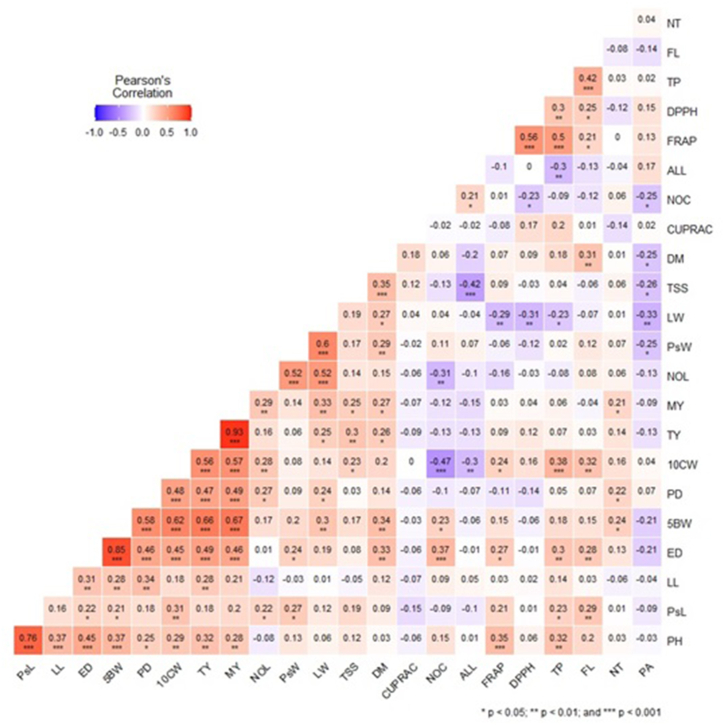
Pearson's correlation coefficient analysis among different morphological and biochemical traits.

The correlation between morphological and biochemical traits was investigated by Pearson correlation analysis (Fig. 1). Plant height was found to be strongly positively correlated with the pseudostem length (r = 0.76) and moderately but significantly with other vegetative traits like leaf length (r = 0.37), bulb equatorial and polar diameter (r = 0.45, r = 0.25), bulb weight and clove weight (r = 0.37, r = 0.29). In this study, the highest correlation was observed between total yield and marketable yield (r = 0.93), and also the average bulb weight correlated positively and very highly significantly with average clove weight (r = 0.62), polar diameter (r = 0.58), marketable yield (r = 0.67) and total yield (r = 0.66). This is expected as a bigger bulb diameter or higher bulb weight can result in a higher yield. Similar findings were reported by Jabbes et al. [65] where they found that the yield was highly correlated with clove weight, bulb weight, and diameter. Singh et al. [66] found that the number of cloves per bulb, weight of cloves, and TSS exerted the highest positive effect on bulb yield per plant. This indicates that the yield potential of garlic is much dependent on its bulb characters and such parameters can be an indicator of plant productivity. However, a low correlation was observed between the number of cloves and the bulb weight (r = 0.23) and also a negative correlation with the total yield (r = −0.13) and marketable yield (r = −0.12) suggesting that the number of cloves do not result in higher bulb weight. This data was also in concurrence with the finding of Benke et al. [67] where no correlation between the average weight of the bulb and the number of clovers per bulb was observed. Plant height was found to be significantly positively correlated with total phenolic content (r = 0.32) and FRAP (r = 0.35) and at a lower correlation with flavonoid content (r = 0.20). A similar correlation was recorded for the pseudostem length with positive but at less significance and lower correlation with total phenolic content (r = 0.23), FRAP (r = 0.21), and flavonoid content (r = 0.29). Phenolic substances have been reported to have effect on the plant growth process that may be divided into three groups i.e., promotive, inhibitory and inactive [68]. Allicin content was found to be in a negative correlation with the vegetative traits and the bulb traits except for the number of cloves where it seems to have a low positive correlation of r = 0.21. Among 29 morphological traits, allicin content was found to be significantly positively associated with pseudostem diameter only but with low correlation coefficient (r = 0.23) [19]. On the other hand, marketable weight, total weight, and clove weight were found to be significantly correlated with total soluble solids (r = 0.27, r = 0.30, r = 0.23, respectively). TSS was recorded to be highly significantly correlated with dry matter (r = 0.35). The dry matter recovery and storage life is associated with TSS value so a higher TSS value will determine higher dry matter recovery. TSS was found to be also associated but not significantly with CUPRAC (r = 0.12), FRAP (r = 0.09), total phenolic content (r = 0.04) but had a negative correlation with DPPH (r = −0.03), and flavonoid content (r = −0.06). Pyruvic acid content had a positive association with DPPH (r = 0.15), FRAP (r = 0.13), and allicin content (r = 0.17). These data can help breeders select target genotypes for developing better varieties with traits for high-yield and health-promoting compounds.

### 3.3 Shannon Diversity index

**Fig. 2:**
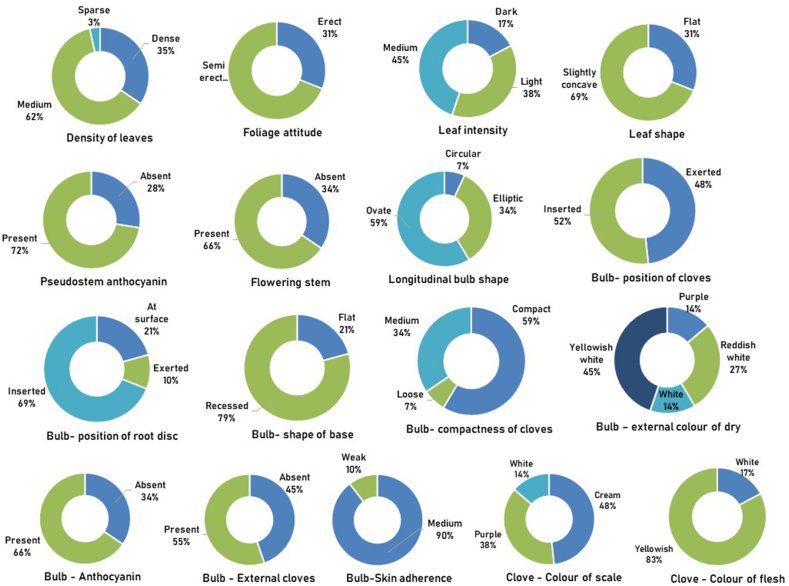
Frequency of garlic genotypes based on the morphological descriptors.

Seventeen morphological descriptors that indicate the stability of traits were observed for the garlic genotypes under study. The diversity index for each descriptor was calculated using the Shannon Diversity index (Table 4, Fig. 2). The diversity index ranged from 0.33 (Bulb-skin adherence of dry external scale) to 1.26 (Bulb-color of dry external scale) indicating variation among the genotypes. Such morphological descriptors can be effectively used in the selection program for the development of improved varieties by identifying and grouping of genotypes.

**Table 4:** Diversity indices of seventeen morphological descriptors in garlic genotypes.

S. No.	Trait	Class or scale of descriptor	Frequency	Relative Frequency (%)	Diversity Index (DI)
1	Density of leaves	Dense	10	34.48	0.78
		Medium	18	62.07	
		Sparse	1	3.45	
2	Foliage attitude	Erect	9	31.03	0.62
		Semi erect	20	68.97	
3	Leaf intensity	Dark	5	17.24	1.03
		Light	11	37.93	
		Medium	13	44.83	
4	Leaf Shape	Flat	9	31.03	0.62
		Slightly concave	20	68.96	
5	Pseudostem anthocyanin	Absent	8	27.58	0.58
		Present	21	72.41	
6	Flowering stem	Absent	10	34.48	0.64
		Present	19	65.52	
7	Bulb shape in longitudinal sect	Circular	2	6.90	0.86
		Elliptic	10	34.48	
		Ovate	17	58.62	
8	Bulb- Position of cloves	Exerted	14	48.28	0.69
		Inserted	15	51.72	
9	Bulb- Position of root disc	At surface	6	20.68	0.82
		Exerted	3	10.34	
		Inserted	20	68.96	
10	Bulb- Shape of base	Flat	6	20.68	0.51
		Recessed	23	79.31	
11	Bulb- Compactness of cloves	Compact	17	58.62	0.86
		Loose	2	6.90	
		Medium	10	34.48	
12	Bulb - Color of dry external scale	Purple	4	13.79	1.26
		Reddish white	8	27.59	
		White	4	13.79	
		Yellowish white	13	44.83	
13	Bulb - Anthocyanin	Absent	10	34.48	0.64
		Present	19	65.52	
14	Bulb – External cloves	Absent	13	44.82	0.69
		Present	16	55.17	
15	Bulb - Skin adherence of dry external scale	Medium	26	89.65	0.33
		Weak	3	10.34	
16	Clove – Colour of scale	Cream	14	48.28	0.99
		Purple	11	37.93	
		White	4	13.79	
17	Clove – Colour of flesh	White	5	17.24	0.46
		Yellowish	24	82.76	
	Mean				0.72
	Maximum				1.26
	Minimum				0.33

**Fig. 3:**
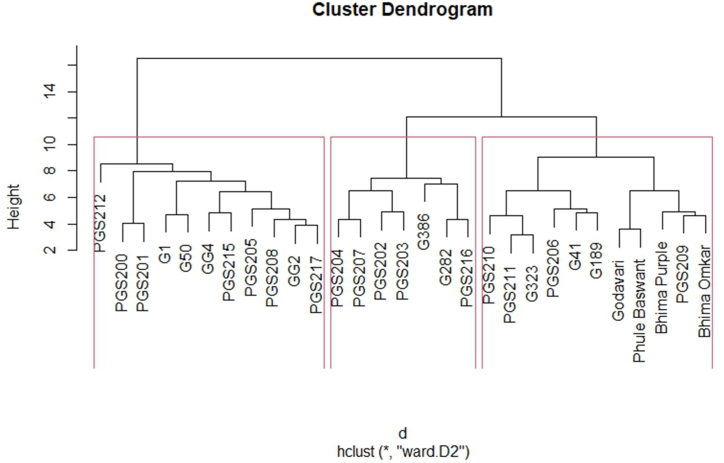
Clustering pattern of garlic genotypes based on morphological and biochemical traits.

Cluster analysis revealed that all the twenty-nine garlic genotypes were clustered into three distinct clusters (Fig. 3). Cluster I was the largest group comprising of 11 genotypes. This cluster comprised of PGS200, PGS201, PGS205, PGS215, PGS212, G1, G50, GG4, PGS208, GG2, PGS217. Cluster II was the smallest cluster comprising of seven genotypes viz., PGS202, PGS203, PGS204, PGS207, G386, G282, and PGS216. This cluster was characterized by the highest plant height, pseudostem length, pseudostem width, leaf length, polar diameter, equatorial diameter, average clove weight, total weight, marketable weight, dry matter content, total phenolics, total flavonoids, FRAP and DPPH antioxidant activity. Hence this group may be used for the selection of genotypes for superior morphological and biochemical traits. Cluster III was the other largest cluster comprising eleven genotypes. This cluster composed of genotypes viz., PGS210, PGS211, G323, PGS206, G41, G189, Godavari, Phule Baswant, Bhima Purple, PGS209 and Bhima Omkar. Clustering was not based on the geographical origin of the genotypes and genotypes from Northern India (NI), Eastern India (EI), Western India (WI), Central India (CI), and North East (NE) were distributed in different clusters. Garlic genotypes were previously classified in a similar way by Panthee et al. [16] where 179 garlic accessions were grouped into three distinct major clusters. Stavělíková [69]. studied 613 garlic genotypes for 22 characters and categorized them into three main groups based on their scape-producing abilities. Similar results have also been reported in garlic genotypes from other studies [21,44,70], and [55].

**Fig. 4:**
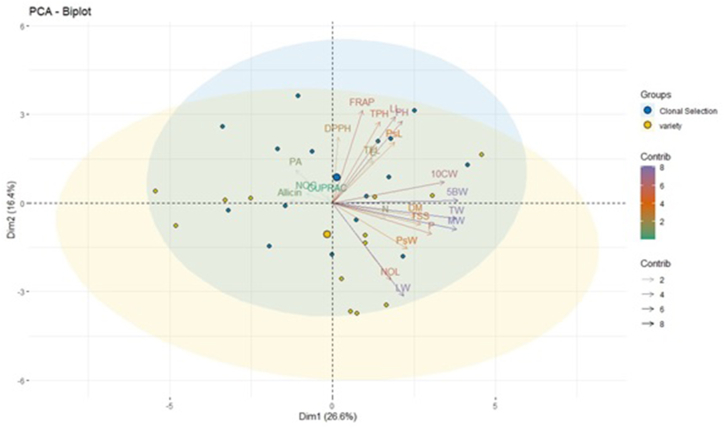
Biplot generated by Principal Component Analysis of 14 morphological and 9 biochemical traits in garlic genotypes.

The biplot was generated from the PCA of the 29 genotypes based on the morphological and biochemical traits. Principal components with eigenvalues lower than 1 were ignored [71]. The data (Table 5, Fig. 4) revealed that 84.01 % variation among the 29 genotypes was explained by the first eight significant principal components. Dim 1 described 26.59 % of the total variance which was mainly contributed by the bulb weight, clove weight, total weight, and marketable weight while negatively loaded with the number of cloves, CUPRAC, Allicin, and Pyruvic acid content. Dim 2 accounted for 42.95 % variation mainly through leaf length, plant height, FRAP, pseudostem length and negatively loaded with pseudostem width, number of leaves, leaf width, polar diameter, TSS, Dry matter, neck thickness, polar diameter, total weight, and marketable weight (Supplementary Table 3). Similar findings have been reported in onion [72] where bulb yield was the major contributor. The positive and negative loading of the traits reflects the positive and negative correlation between the components and variables. Hence, one important variable from the identified groups can be selected for a targeted improvement program.

**Table 5:** Eigen-value and contribution of the principal component axes towards total genetic variation in garlic genotypes under study.

DIM	Eigenvalue	Variability %	Cumulative %
DIM1	6.117	26.596	26.596
DIM2	3.763	16.361	42.958
DIM3	2.361	10.265	53.223
DIM4	1.976	8.591	61.815
DIM5	1.562	6.792	68.608
DIM6	1.435	6.241	74.849
DIM7	1.074	4.672	79.522
DIM8	1.034	4.496	84.018
DIM9	0.774	3.366	87.384
DIM10	0.642	2.795	90.180
DIM 11	0.510	2.221	92.401
DIM12	0.430	1.869	94.271
DIM13	0.329	1.434	95.706
DIM14	0.287	1.247	96.953
DIM15	0.219	0.955	97.908
DIM16	0.162	0.705	98.614
DIM17	0.105	0.456	99.071
DIM18	0.093	0.405	99.476
DIM19	0.067	0.291	99.768
DIM20	0.027	0.118	99.887
DIM21	0.011	0.047	99.935
DIM22	0.008	0.035	99.970
DIM23	0.006	0.293	100.000

### 3.4 Molecular characterization

**Fig. 5:**
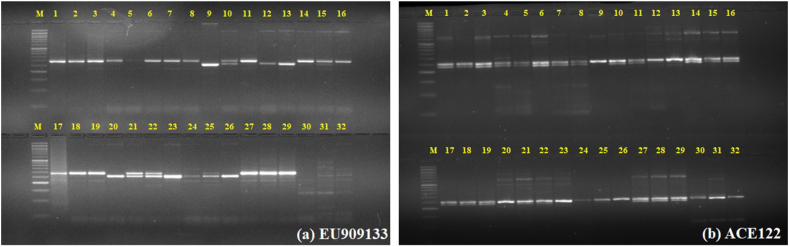
Gel profiling picture of SSR markers (a) EU909133 and (b) ACE122 M = DNA ladder 50 bp.

In this study, a total of 214 SSR markers were screened across 29 diverse garlic genotypes and 3 outgroup species i.e., *A.cepa* L.*, A.fistulosum* L. and *A. tuberosum* L. Out of 214, nine polymorphic SSR markers viz., EU909133, ACE122, ACM091, AsESSR47, AsESSR82, AsESSR33, AsESSR91, AsESSR103, and AsESSR78 showed good amplification and the ability to identify high levels of polymorphism. A total of 33 SSR alleles were amplified from the DNA of 29 genotypes and the 3 outgroup species. The number of alleles detected by these SSR markers varied from as few as two (ACM091, AsESSR91, AsESSR103) to six alleles (AsESSR47) with an average of 3.67 alleles (Table 6, Fig. 5). Thirty-nine bands ranging from two to eight bands with a mean value of 3.9 bands per primer pair amplified by 10 SSR primer pairs in 53 accessions were reported by Kumar et al. [73]. Kiraç et al. [74] also obtained 47 polymorphic bands using 10 ISSR markers in 39 garlic genotypes of Turkey. On the other hand, Chen et al [75]. reported a lower mean effective number of alleles of 1.47 (range 1.27–1.77) in 3 garlic accessions using 10 SSRs. Barboza et al. [76] using 10 ESSR markers in 73 garlic accessions reported 43 alleles amplified with an average of 4.3 alleles (range 3–7) which were higher than our results. The markers discriminatory power estimated by the Polymorphic information content (PIC) varied from 0.210 (AsESSR103) to 0.730 (AsESSR78) with an average of 0.497. Four SSR markers viz., ACE122, ACM091, AsESSR91, and AsESSR103 had PIC values less than 0.5 (Table 6). This level of polymorphism is lower than the mean PIC reported by Da Cunha et al. [77], where they observed a range of 0.16–0.75 in 136 garlic accessions utilizing 17 gSSR markers. Higher PIC values were also reported by other authors [75] (PIC of 0.72) [78], (PIC of 0.63) and [79] (PIC of 0.60).

**Table 6:** Amplification range (bp), annealing temperature, number of alleles and PIC of each marker.

Marker	Amplification (bp)	Annealing temperature (°C)	Number of Alleles	PIC
EU909133	250–297	56	4	0.596
ACE122	235–265	52	5	0.438
ACM091	180–200	52	2	0.314
AsESSR47	650–700	60	6	0.721
AsESSR82	360–450	59	4	0.695
AsESSR33	230–270	59	3	0.523
AsESSR91	300–350	59	2	0.245
AsESSR103	270–300	59	2	0.210
AsESSR78	270–310	59	5	0.730
Mean			3.67	0.497

**Fig. 6:**
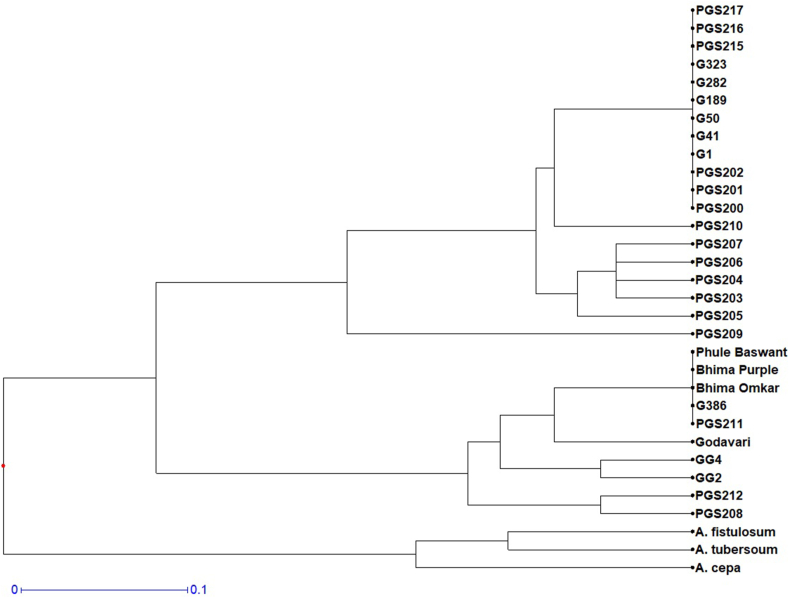
Clustering pattern of garlic genotypes based on SSR markers using UPGMA.

The twenty-nine genotypes and three species were found to be classified into four main clusters based on the cluster analysis (Fig. 6). Cluster I, was the largest group comprising 18 genotypes (PGS200, PGS201, PGS202, PGS203, PGS204, PGS205, PGS206, PGS207, PGS210, PGS215, PGS216, PGS217, G1, G41, G50, G189, G282, G323). Cluster II was composed of a single genotype (PGS-209). Cluster III had 10 genotypes (PGS208, PGS211, PGS212, G386, Bhima Omkar, Bhima Purple, Phule Baswant, Godavari, GG2, GG4). Three species formed an outgroup (*A.cepa* L.*, A.fistulosum* L.*, A. tuberosum* L.) and were grouped into cluster IV. In Cluster I, all the genotypes of the North Eastern (NE) region, maximum genotypes of North India (NI), and some genotypes of Eastern India (EI), Southern India (SI), and Western India (WI) clustered together. It was surprising to see that all the commercial varieties released by NHRDF, except G386, showed a very high degree of similarity and clustered together. This may be due to the reason that the initial material for all the varieties may be similar. Further studies with more SSR markers distributed on different chromosomes of the garlic genome will help us to reveal the true genetic identity of the varieties and other breeding material. In Cluster II, all genotypes of Central India (CI) viz., PGS211, PGS212) and most of the genotypes of EI and WI clustered together. All the commercial varieties from Eastern and Western India clustered together in this group. In cluster III, only one genotype from Western India (WI) was positioned. This indicates that the clustering was based on geographical origin in this study. Research findings associating genetic clustering with geographical locations have been reported by other authors [33,79]. Contrary to our findings, genetic clustering based on flowering behavior has also been reported in the literature [12,13,20,80].

It was observed that the clustering based on morpho-biochemical characterization was influenced by the traits and did not reveal the clustering based on geographic origin whereas molecular characterization clustered the genotypes based on geographical origin. Various studies have shown no agreement between clustering based on molecular markers and agromorphological traits [[81], [82], [83]]. There are many reasons attributed to this disagreement which may be that the molecular markers used may not be associated with the gene involved in agro-morphological traits or the material may be same but cultivation over the years and transfer to different places may have led to different naming by farmers at other locations. Clustering based on molecular markers revealed that most of the commercial varieties released by NHRDF are very similar to each other whereas based on morpho-biochemical characterization, all the varieties of NHRDF were distributed in different clusters. PGS209 clustered separately in the SSR characterization which may or may not be true and needs further investigation. Garlic is a vegetatively propagated crop with a huge genome size and a large number of SSR markers distributed uniformly on all the chromosomes are required to reveal the true genetic diversity.

## 4 Conclusions

In this study, a three-way approach to determine the genetic variability among the garlic genotypes was carried out. Significant variation for agro-morphological and biochemical traits was observed indicating good scope of improvement. Higher PCA for morphological traits revealed that environment plays a significant part in trait expression in garlic. Correlation studies revealed significant relationship between morphological and biochemical traits. Cluster analysis, based on morphological and biochemical characters, revealed three clusters and clustering was not based on geographical origin. Molecular markers clustered genotypes on the basis of geographical origin and genotypes with maximum similarity were also observed. Hence, molecular markers should be used to reveal an unbiased estimate of grouping garlic genotypes. Very few (4.2 %) SSR markers were polymorphic which leads to the demand of mining more SSRs from the already available genomic resources in garlic so that the total diversity of garlic genotypes is revealed in a systematic way. This will lead to the development of superior garlic varieties and identification of duplicates for efficient germplasm management.

## Data availability statement

All data generated or analyzed during this study are included in this published article.

## CRediT authorship contribution statement

**Karishma Pasupula:** Writing – original draft, Investigation. **Priyanka Verma:** Validation, Supervision, Methodology. **Masochon Zimik:** Writing – original draft, Formal analysis, Data curation. **Charanjit Kaur:** Visualization, Resources. **Sujata Vasudev:** Writing – review & editing, Resources. **Anil Khar:** Writing – review & editing, Supervision, Project administration, Investigation, Formal analysis, Conceptualization.

## Declaration of competing interest

The authors declare that they have no known competing financial interests or personal relationships that could have appeared to influence the work reported in this paper.
