# Supplementary material for: Morphological, biochemical and molecular characterization of short-day tropical Indian garlic (Allium sativum L.)
Source: Heliyon. 2024 Sep 6;10(18):e37553. doi: 10.1016/j.heliyon.2024.e37553 (PMC11414556; doi:10.1016/j.heliyon.2024.e37553)
Supplement: Multimedia component 1 [file mmc1.docx]

**Table 1. List of genotypes used for morphological, biochemical and molecular analysis**

| **S. No.** | **Genotype** | **Source** | **Status** | **Latitude** | **Longitude** | **Releasing Institute** | **Remarks** |
| --- | --- | --- | --- | --- | --- | --- | --- |
| 1 | PGS200 | Arunachal Pradesh (NE) | L | 27°21'55.2" | 93°02'35.6" | - | – |
| 2 | PGS201 | Assam (NE) | L | 26°54.'07.2" | 93°43'0.5" | - | - |
| 3 | PGS202 | Assam (NE) | L | 26°54'07.2" | 93°43'0.5" | - | - |
| 4 | PGS203 | Assam (NE) | L | 27°7'26.9" | 93°51'16.0" | - | - |
| 5 | PGS204 | Arunachal Pradesh (NE) | L | 27°07'43.3" | 93°44'33.1" | - | - |
| 6 | PGS205 | Arunachal Pradesh (NE) | L | 27°07'43.3" | 93°44'33.1" | - | - |
| 7 | PGS206 | Rajasthan (NI) | CS | - | - | - | 4th generation clonal selection of local material from Rajasthan |
| 8 | PGS207 | Rajasthan (NI) | CS | - | - | - | 4th generation clonal selection of local material from Rajasthan |
| 9 | PGS208 | Rajasthan (NI) | CS | - | - | - | 4^th^ generation clonal selection of local material from Rajasthan |
| 10 | PGS209 | Gujarat (WI) | CS | - | - | - | 4^th^ generation clonal selection from Gujarat |
| 11 | PGS210 | Gujarat (WI) | CS | - | - | - | 4^th^ generation clonal selection from Gujarat |
| 12 | PGS211 | Madhya Pradesh (CI) | CS | - | - | - | 3rd generation clonal selection from Madhya Pradesh |
| 13 | PGS212 | Madhya Pradesh (CI) | CS | - | - | - | 3rd generation clonal selection from Madhya Pradesh |
| 14 | PGS215 | Ladakh (NI) | CS | MKG87 | - | - | 3^rd^ generation clonal selection from local material from Ladakh |
| 15 | PGS216 | Uttar Pradesh (NI) | CS | ACC40 | - | - | 3^rd^ generation clonal selection from local material from Uttar Pradesh |
| 16 | PGS217 | Delhi (NI) | CS | PGS105 | - | - | 5^th^ generation clonal selection from local material from Delhi |
| 17 | G1 | Delhi (NI) | CV | - | Yamuna Safed | NHRDF, Nasik |  |
| 18 | G41 | Bihar (EI) | CV | - | Agrifound White | NHRDF, Nasik |  |
| 19 | G50 | Haryana (NI) | CV | - | Yamuna Safed-2 | NHRDF, Nasik |  |
| 20 | G189 | Haryana (NI) | CV | - | Yamuna Safed-5 | NHRDF, Nasik |  |
| 21 | G282 | Tamil Nadu (SI) | CV | - | Yamuna Safed-3 | NHRDF, Nasik |  |
| 22 | G323 | Uttar Pradesh (NI) | CV | - | Yamuna Safed-4 | NHRDF, Nasik |  |
| 23 | G386 | Uttar Pradesh (NI) | CV | - |  | NHRDF, Nasik |  |
| 24 | GG2 | Gujarat (WI) | CV | - | Gujarat Garlic 2 | JAU, Junagarh |  |
| 25 | GG4 | Gujarat (WI) | CV | - | Gujarat Garlic 4 | JAU, Junagarh |  |
| 26 | Bhima Omkar | Bihar (EI) | CV | - |  | DOGR, Pune |  |
| 27 | Bhima Purple | Orissa (EI) | CV |  | - | DOGR, Pune |  |
| 28 | Godavari | Maharashtra (WI) | CV |  | - | MPKV, Rahuri |  |
| 29 | Phule Baswant | Maharashtra (WI) | CV |  | - | MPKV, Rahuri |  |

NE-North East India; EI-Eastern Indian; WI-Western India; CI=Central India; NI-Northern India; SI-Southern India

L-Landrace; CS=Clonal Selection; CV=Commercial Variety

**Table 2. Microsatellites (SSRs) showing polymorphism in garlic accessions**

| **Locus** | **Sequence SSR** | **Forward primer (5’-3’)** | **Reverse Primer (5’-3’)** | **Reference** |
| --- | --- | --- | --- | --- |
| GB-ASM-040 (EU909133) | (AC)6, (AC)14-(AT)15 | CACAGCAACATGCACCAT | TGCCGGAACTCGATATT | Ma *et al.* 2009 |
| ACE122 | (TCT)9(TC)3 | ACCCATGCCTTCCATTTCACTT | TTTAGAAGACCGATTCCAGGCAAAC | Tsukazaki *et al*. 2010 |
| ACM091 | (TCT)10 | TCTCCTCCTCTAACCAGCCA | GGTGCTCCAGTTGAGCTTTC | Jakše *et al*. 2005 |
| AsESSR33 | (TTC)5 | CCTCCTCTCCTCCTCCATCT | AAGTCTGCGGCTGATACGTT | Barboza *et al*. 2018 |
| AsESSR47 | (AGC)5 | CGTCCGCCAATTGAATACTTA | ACAACCTGCTGCTATGGTCAC | Barboza *et al*. 2018 |
| AsESSR78 | (CTTTT)3 | TCTGACAGACGACCTGGAGAT | ATCACTGCCTCTCCACAAGAA | Barboza *et al*. 2018 |
| AsESSR82 | (AACGGC)4 | ACTCCCTCTCCAACTTCCAAC | CAAGGGTTACAAAGTCGCTGA | Barboza *et al*. 2018 |
| AsESSR91 | (CAAATC)4 | GTTCTCCGTTGCGTCAATC | GAATTTGCATCTTTCCCCTTC | Barboza *et al*. 2018 |
| AsESSR103 | (TCC)6(TTC)5 | TTATCTCCGTCGACCCTTTC | GAACGGAGGAGAGAGAGGAGA | Barboza *et al*. 2018 |

**Table 3. Contribution of different morpho-biochemical traits of onion towards the major principal components**

|  | Dim.1 | Dim.2 | Dim.3 | Dim.4 | Dim.5 |
| --- | --- | --- | --- | --- | --- |
| PH | 0.489 | 0.639 | 0.304 | -0.273 | 0.218 |
| PsL | 0.434 | 0.474 | 0.106 | -0.247 | 0.444 |
| PsW | 0.524 | -0.354 | 0.395 | 0.210 | 0.333 |
| NOL | 0.410 | -0.597 | -0.365 | 0.101 | 0.376 |
| LL | 0.440 | 0.670 | 0.369 | -0.197 | -0.067 |
| LW | 0.500 | -0.722 | 0.233 | 0.139 | 0.139 |
| P | 0.698 | -0.238 | -0.190 | -0.416 | 0.150 |
| E | 0.287 | 0.330 | 0.250 | 0.288 | -0.112 |
| N | 0.375 | -0.113 | 0.122 | -0.471 | -0.351 |
| ABW | 0.883 | 0.021 | 0.273 | -0.058 | -0.093 |
| NOC | -0.184 | 0.161 | 0.893 | 0.118 | -0.151 |
| 10CW | 0.789 | 0.031 | -0.441 | -0.107 | 0.028 |
| TSS | 0.623 | -0.164 | 0.039 | 0.284 | -0.475 |
| TPC | 0.330 | 0.631 | -0.161 | 0.230 | 0.053 |
| TFC | 0.277 | 0.345 | -0.146 | 0.375 | 0.605 |
| CUPRAC | -0.039 | 0.053 | -0.073 | 0.529 | -0.171 |
| FRAP | 0.209 | 0.721 | -0.081 | 0.160 | -0.169 |
| DPPH | 0.039 | 0.514 | -0.426 | 0.336 | -0.091 |
| Allicin | -0.299 | 0.016 | 0.365 | -0.272 | 0.277 |
| PA | -0.258 | 0.254 | -0.435 | -0.449 | -0.153 |
| DM | 0.585 | -0.099 | 0.062 | 0.466 | -0.052 |
| TW | 0.874 | -0.121 | -0.105 | -0.085 | -0.200 |
| MW | 0.874 | -0.201 | -0.107 | -0.150 | -0.211 |

PH-Plant height; PsL-Pseudostem length; PsW-Pseudostem width; NOL-Number of leaves; LL-Leaf length; LW-Leaf width; P-Polar diameter; E-Equatorial diameter; N-Neck thickness: ABW-Average bulb weight; NON-Number of cloves; 10CW-10 clove weight; TSS-Total soluble solids; TPC-Total phenolic content; TFC-Total flavonoid content; PA-Pyruvic acid; DM-Dry matter, TW-Total weight; MW-Marketable weight
